# Sedentariness and Urinary Metabolite Profile in Type 2 Diabetic Patients, a Cross-Sectional Study

**DOI:** 10.3390/metabo10050205

**Published:** 2020-05-18

**Authors:** Elisa Benetti, Erica Liberto, Davide Bressanello, Valentina Bordano, Arianna C. Rosa, Gianluca Miglio, Jonida Haxhi, Giuseppe Pugliese, Stefano Balducci, Chiara Cordero

**Affiliations:** 1 Department of Drug Science and Technology University of Turin, 10125 Turin, Italy; erica.liberto@unito.it (E.L.); davide.bressanello@unito.it (D.B.); valentina.bordano@unito.it (V.B.); ariannacarolina.rosa@unito.it (A.C.R.); gianluca.miglio@unito.it (G.M.); chiara.cordero@unito.it (C.C.); 2 Department of Clinical and Molecular Medicine, ‘‘La Sapienza” University, 00183 Rome, Italy; efijona@yahoo.com (J.H.); giuseppe.pugliese@uniroma1.it (G.P.); s.balducci@hctdiabete.it (S.B.); 3 Diabetes Unit, Sant’Andrea Hospital, 00183 Rome, Italy; 4 Metabolic Fitness Association, Monterotondo, 00015 Rome, Italy

**Keywords:** sedentariness, urine metabolome, type 2 diabetes, observational study, comprehensive two-dimensional gas chromatography, dual parallel detection by mass spectrometry and flame ionization detector, chemometrics

## Abstract

Recent findings indicate a significant association between sedentary (SED)-time and type 2 diabetes mellitus (T2DM). The aim of this study was to investigate whether different levels of SED-time could impact on biochemical and physiological processes occurring in sedentary and physically inactive T2DM patients. In particular, patients from the “Italian Diabetes and Exercise Study (IDES)_2 trial belonging to the first and fourth quartile of SED-time were compared. Urine samples were analyzed by comprehensive two-dimensional gas chromatography (GC × GC) with parallel detection by mass spectrometry and flame ionization detection (GC × 2GC-MS/FID). This platform enables accurate profiling and fingerprinting of urinary metabolites while maximizing the overall information capacity, quantitation reliability, and response linearity. Moreover, using advanced pattern recognition, the fingerprinting process was extended to untargeted and targeted features, revealing diagnostic urinary fingerprints between groups. Quantitative metabolomics was then applied to analytes of relevance for robust comparisons. Increased levels of glycine, L-valine, L-threonine, L-phenylalanine, L-leucine, L-alanine, succinic acid, 2-ketoglutaric acid, xylitol, and ribitol were revealed in samples from less sedentary women. In conclusion, SED-time is associated with changes in urine metabolome signatures. These preliminary results suggest that reducing SED-time could be a strategy to improve the health status of a large proportion of diabetic patients.

## 1. Introduction

Type 2 diabetes mellitus (T2DM) is one of the most widespread diseases in the world [1]. It is a complex metabolic disorder with multiple causes and there is compelling evidence which indicates that a physically active lifestyle can reduce cardiovascular risk factors and improve glycemic control and well-being in people with T2DM [2,3,4,5,6,7]. Physical activity (PA) is considered a cornerstone of the prevention and management of this disease. However, a significant association between sedentary (SED)-time and metabolic risk was recently observed in T2DM patients, independently of several confounders, including time spent in moderate-to-vigorous-intensity PA (MVPA). Indeed, after accounting for SED-time, MVPA was not associated with clustered metabolic risk [8,9]. These data are consistent with previous reports showing that daily sitting time or television viewing were positively associated with all-cause and cardiovascular mortality [10,11], as well as cardiovascular risk factors [12,13]. Taken together, these observations support the concept that physical inactivity and sedentary behavior negativelyaffect health status through distinct pathways thus highlighting the need to reduce SED-time in addition to increasing PA.

Notably, SED-time is not related to the time spent in PA. Indeed, the term “sedentary behavior” differs from “physical inactivity” [14]. In keeping with the Sedentary Behavior Research Network, sedentary behavior is defined as “any waking behavior characterized by an energy expenditure ≤1.5 METs (multiples of the resting metabolic rate, defined as an oxygen uptake of 3.5 mL/kg/min) while in a sitting or reclining posture” [15,16], whereas the term “physical inactivity” refers to not achieving the minimum recommendations of moderate-to-vigorous physical activity [17].

Although the majority of epidemiological studies have focused on the effect of PA, rather than on the reduction of SED-time, theattention on the deleterious consequences of prolonged SED-time has been recently intensified, with strong evidence linking SED-time to an increased risk of T2DM development [18,19]. Previous studies haveshown a positive correlation between SED-time and high glucose levels [20] or insulin resistance [8], andthat interrupting prolonged sitting in T2DM people improved the glycemic control [21,22].

One comprehensive approach to evaluate the systemic metabolism, reflecting the metabolic health of an individual, is using metabolomics. Its importance to evaluate early changes in metabolite profiles related to metabolic disease, in particular diabetes [23,24] is well known. Interestingly, more recently, metabolomics have been also adopted to identify pathways involved in the beneficial effects evoked by physical activity [25,26,27,28]. In contrast, very limited data are available on SED-time and its potential ability to affect the metabolic fine print (i.e., fingerprint) in biofluids [29]. In particular, to the best of our knowledge, the effect of SED-time has been not investigated in urine metabolomic profiles.

Within this challenging context, this study on urinary metabolic signatures could provide some insight on the mechanisms underlying the impact of sedentary behavior on systemic metabolism, and provide further information on individual response [30,31,32]. To accurately track changes and/or to monitor the evolution of primary metabolites in urine samples, comprehensive two-dimensional gas chromatography coupled with mass spectrometry (GC × GC-MS) represents one of the most advanced and informative GC platforms currently available [33]. Its separation power, sensitivity, and structured bi-dimensional (2D) patterns generated for chemically correlated compounds are fundamental keyfeatures at the basis of its information potential for complex biological samples’ profiling and fingerprinting [30,33,34,35].

For this study, a GC × GC system with parallel dual secondary column-dual detection configuration (GC × 2GC-MS/FID) was adopted to maximize the overall information capacity [36,37] by improving quantitation reliability and response linearity. In addition, advanced pattern recognition methodologies based on the 2D data were employed, extending the investigation to untargeted and targeted 2D-peak features for the most inclusive fingerprinting (i.e., *UT fingerprinting*) [30,38].

This study aimed to investigate whether in sedentary and physically inactive patients with T2DM, different levels of SED-time could affect the urinary metabolic signatures in order to better understand the biological pathways potentially linking the prolonged SED-time to T2DM. To this purpose, we selected a sub-group of T2DM patients enrolled in the Italian Diabetes and Exercise Study (IDES)_2 study [39] and examined urine samples using the following procedure.

## 2. Results

### 2.1. Characteristics of Study Patients

The study population was a subgroup (n = 86) of T2DM patients previously enrolled in the IDES_2 clinical trial [39] and was separated into two different groups: Group 1, the less sedentary group, belonging to the first quartile of SED-time; Group 2, the more sedentary group, belonging to the fourth quartile of SED-time (Table 1, Table S1). Demographic and clinical parameters of the two selected study population groups are reported in Table 2.

Significant differences between the groups were observed for age and, as it could be expected, for BMI, triglycerides, systolic blood pressure, and cardiovascular risk scores. Within the same gender (female 1 vs. female 2 or male 1 vs. male 2, Table S2) differences between the two groups for age and cardiovascular risk scores in both males and females were observed, whereas triglycerides and blood pressure were different just within group for women.

### 2.2. Urinary Metabolic Signatures: Bottom-Down Approach for Target Candidates’ Selection

**Figure 1:**
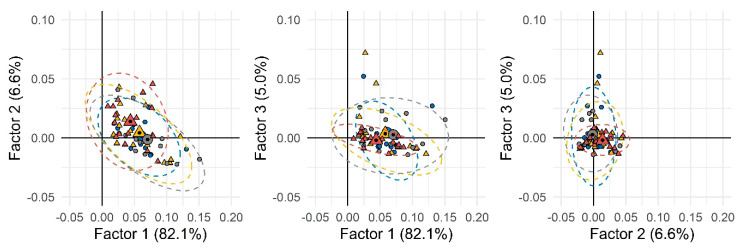
Principal component analysis (PCA) scores-plot based on all detected peak-region features over the entire sample set. Group 1 (less sedentary group) and Group 2 (more sedentary group) were depicted as circles and triangles, respectively. Different colors were used to indicate males (blue and yellow) and females (gray and dark red). Small and large (with a black spot) symbols represent individual and mean values, respectively.

The first step of the data elaboration work-flow based on the chemical/metabolic fingerprints aimed at delineating, if possible, natural groups’ conformation within the set of individuals’ urinary signatures. A first elaboration was performed by considering all available samples, including analytical replicates, and for each sample, all untargeted and targeted peak-regions (UT) corresponding to all detected compounds reliably matching across all 2D patterns. Data normalization was by ISTD stand total analytical response. Analytes’ quantitative descriptors were thus 2D-nomalized volumes % (i.e., 2D peak volumes divided by ISTD response and subsequently normalized over the total analytical response %). The resulting data matrix was 152 × 850 (samples × UT features). When submitted to unsupervised PCA (principal component analysis), the resulting score-plot (Figure 1) did not show any clear clustering between the two groups, suggesting the presence of several confounding variables not strictly related to the phenomenon under study (i.e., SED-time).

Thereby, as second step, a supervised approach through orthogonal partial least square-discriminant analysis (OPLS-DA) was applied on all UT peak-features to see the differential effect of sedentariness on genders. OPLS-DA correlation spectrum showed a positive correlation between each variable in the x block (untargeted peak-features) with y variables (samples grouped in gender and treatment). With this function, the graphical exclusions of poorly correlating x variables was facilitated.

**Figure 2:**
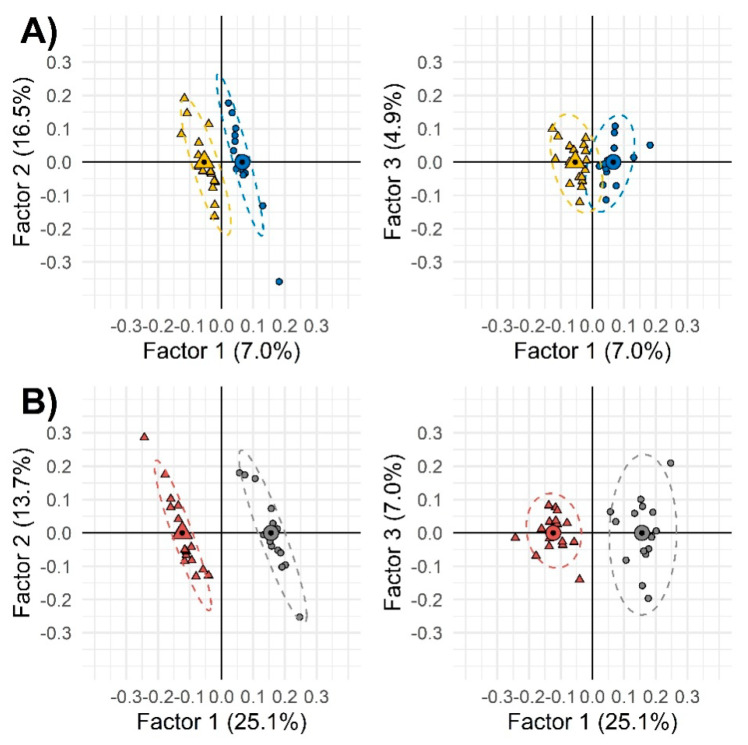
Two-by-two orthogonal partial least square-discriminant analysis (OPLS-DA) of the combined untargeted and targeted UT peak-region features filtered by Fisher ratio from the whole samples’ set. Group 1 (less sedentary group) and Group 2 (more sedentary group) were depicted as circles and triangles, respectively. Different colors were used to indicate males (panel (**A**), blue and yellow) and females (panel (**B**), gray and dark red). Small and large (with a black spot) symbols represent individual and mean values, respectively.

The aim of this supervised exploration was to better outline those variables mostly related to the treatment, while reducing the noise from confounding factors, rather than to develop a classification model. The sample set was not sufficient to perform a fullvalidation and the risk of overfitting was expected. However, the strategy allowed effective selection of variables, i.e., targeted analytes to be quantified with suitable accuracy and precision by GC-MS. Results, shown in Figure 2, suggest that among females, as compared with males, the effect of sedentariness has a greater impact on urinary metabolic signatures.Model performances referred of: (1) 94% accuracy for females and 100% for males, (2) 93.8% sensitivity for females and 100% for males, and (3) 100% specificity for both gender groups.

Results from OPLS-DA correlation spectra enabled feature selection toward target analytes that were thus submitted, in the third step, to quantitative metabolomics by GC-MS. Quantitation was also extended to some additional metabolites selected after a careful survey of the scientific literature within those metabolic markers with relevant information potential.

Quantitative results, normalized over creatinine concentration, are reported as supplementary information (Table S3). Data were submitted to supervised linear discriminant analysis (LDA) to facilitate the evidence of meaningful variables between groups and for each gender. Table 3 reports metabolites percentage of variation referred to the median relative increment/decrement of the Group 1 vs. Group 2.

In females belonging to the less sedentary group (Group 1), it was observed a relevant up-regulation of several urinary metabolites: Glycine (^§^*p* = 0.011), L-alanine (*p* = 0.042), 2-ketoglutaric acid (*p* = 0.011), L-threonine (^§^*p* = 0.041), L-valine (^§^*p* = 0.029), succinic acid (*p* = 0.031), L-phenylalanine (*p* = 0.014), L-leucine (*p* = 0.024), xylitol (*p* = 0.015), and ribitol (*p* = 0.018). The information power of the selected metabolites was assessed by Kruskal–Wallis test followed by the Dunn’s comparison of the mean (*p*) and Bonferroni correction (^§^*p*).

## 3. Discussion

In this study patients were evaluated by monitoring the relationship between SED-time and urinary metabolome. An initial analysis of our data using PCA revealed no clearly distinguishable differences in urine metabolic signature in relation to SED-time. However, LDA analysis showed some differences between the two groups, in particular among females, who appeared more responsive than males to the different levels of SED-time. This observation is not surprising [40] since previous studies also showed more pronounced plasma metabolite changes correlated to physical activity among females rather that in males [25,26]. Although previous studies have evaluated the effects of SED-time on plasma metabolomic profiles [26,41], this study presents for the first time an observed effect of SED-time on urine metabolites and attempts to give a clinical meaning to the relationship between reduced SED-time and T2DM.

In order to evaluate whether metabolite changes observed in less sedentary diabetic women could affect their health status, our results attempted to correlate metabolomic analysis to T2DM.

Xiao et al. and Fukai et al. have shown a lower aminoacids plasma level in people at the lower level of sedentariness. Although insulin resistance and diabetes development show a positive correlation with high plasma levels of branched chain amino acids (BCAAs, valine, leucine, isoleucine) [42,43], the causal link is still under debate [44]. Several groups have provided mechanistic explanations for this correlation. In particular, insulin resistance is evoked by an excess of the catabolic flux of BCAA, rather than theplasma level [45]. This data is supported by Menni C. et al. [46], according to whom diabetes is more related tothe breakdown of BCAA, rather than the elevated level of these aminoacids.

The first step of BCAA catabolic pathways is the reversible transamination reaction that converts them into branched-chain ketoacids. The pair glutamate/2-ketoglutarate is necessarily involved in this process. Indeed, BCAA transfer the amino group to 2-ketoglutarate, thus converting it into glutamate [47]. Interestingly, our data showed a higher level of 2-ketoglutaric acid in the urine of the less sedentary women, thus allowing us to hypothesize a lower level of BCAA catabolism in these patients. As BCAA catabolism is positively correlated to insulin resistance, this data could indicate anincreased insulin sensitivity in less sedentary women. In support of this observation, 2-ketoglutaric acid was also recently indicated as a urine marker of T2DM. Indeed, lower concentrations of this metabolite are found in urine samples of diabetics compared to healthy people [48]. Similar results were also seen in preclinical studies [49].

Few data are reported in the literature on the level of aminoacids in the urine of diabetic patients. Salek et al. [50] showed a decrease in the excretion of several aminoacids, including valine and leucine, in the urine of diabetic patients. Interestingly, an increase of amino acid concentration in the urine has been also observed in diabetic patients following rosiglitazone treatment [51], thus suggesting that this change could be associated to the pharmacological treatment that, by reducing insulin resistance, also decreases the demand for amino acids as substrates for gluconeogenesis. Therefore, the increased aminoacid excretion observed in less sedentary women could be suggestive of a minor need of glucose synthesis and, accordingly, improved insulin sensitivity.

In comparison to healthy subjects, T2DM patients exhibit a decreased excretion of some intermediates of the tricarboxylic acid (TCA) cycle, which could indicate a mitochondrial dysfunction [51,52,53]. Interestingly, in our study we observed a higher level of succinic acid, as well as 2-ketoglutaric acid, in the urine of females belonging to the lower sedentary group. This data possibly indicate that SED-time reduction could exert a positive effect on the health status.

We also found ameaningful increase in glycine level in the urine of the femalesin the lower sedentary group. Glycine could result from the action of the alanine-glyoxylate aminotransferase-2 (AGT-2) on its substrate glyoxylate [54]. AGT-2 has been shown as having an important action in the blood pressure regulation [55]. Indeed, a suppression of the activity of this enzyme could be associated with hypertension and increased glyoxylate levels [56]. In contrast, by observing increased level of glycine in the urine in less sedentary women, we can speculate that the activity of this enzyme is higher in these patients. Notably, in keeping with these observations, as reported in Table S2, less sedentary females presented a significantly lower blood pressure value (*p* < 0.050) in comparison with more sedentary women. The increased glycine excretion could contribute, at least in part, to this effect.

Overall, our urinary metabolomic data allowed us to hypothesize that SED-time reduction may exert a positive effect on the health status. Thus, we could suggest that limiting SED-time could be a useful strategy in particular for females, the group that has been demonstrated to be an independent predictor of failure to achieve therapeutic targets in diabetic patients [57].

This study had some limitations. First, we cannot exclude the possibility that the significant difference in terms of age, light PA, and MVPA levels between the two groups could have affected our results. Second, the cross-sectional nature of our study did not allow us to formulate causal relationship. However, our promising findings may inform future perspective investigations on the effect of SED-time reduction especially in longitudinal studies, which might lead to better understand the clinical meaning of this strategy and infer more solid conclusions. Finally, our results indicated that SED-time could affect the concentration of urinary metabolites, in particular in females, the most responsive gender, suggesting that lower level of SED-time could be associated to better T2DM outcome. Whether our data will be confirmed by ad hoc longitudinaltrials, SED-time reduction could become a positive and intriguing opportunity for diabetic patients. Indeed, it is well known that increases of physical activity lead to major beneficial effects in these patients, but unfortunately, compliance is quite low. The possibility to achieve significant results with minimal efforts could encourage the patients to improve their adherence to the lifestyle indications suggested by physicians, with consequently better outcomes.

## 4. Materials and Methods

### 4.1. Study Population

Study population included men and women from the IDES_2 trial [39]. We selected a subgroup of physically inactive and sedentary patients (n = 86) in order to compare patients at the first quartile of SED-time (n = 43) towards those at the fourth quartile (n = 43). The main entry criterion was T2DM (defined by the ADA criteria [58]) of at least one-year duration. Additional requirements were: Age 40–80 years, body mass index 27–40 kg/m^2^ (i.e., insufficient amounts of PA according to current guidelines [59]) and sedentary lifestyle (i.e., > 8 h/day [60]) from at least 6 months, ability to walk 1.6 km without assistance, and eligibility after cardiologic evaluation. At inclusion in the study T_0_, patients’ 24-hurine was collected and conserved at –20°C until analysisas baseline data.

All subjects gave informed consent for inclusion before participation in the study IDES_2. This study was conducted in accordance with the Declaration of Helsinki, and the protocol was approved by the ComitatoEticodell’AziendaOspedalieraSant’Andrea Prot. n. 212/2012 (ClinicalTrials.gov; NCT01600937; 10 October 2012).

#### Measurements of Sedentariness

At baseline, MVPA, LPA, and SED-time were measured by the use of an accelerometer (MyWellness Key, Technogym, Cesena, IT), which was validated versus Actigraph [61] and provided accurate measures of the minutes spent at different intensities and the total PA volume [6], even in individuals with T2DM [62]. A daily diary was kept for reporting the time spent wearing the instrument, sleeping and napping, and performing non-accelerometer recordable PAs such as swimming, cycling, skiing, etc. Participants were asked to attach the device at the waist and to wear it all day (except if swimming) up to bedtime, in order to avoid the influence of the “time accelerometer worn”, which may cause underestimation of daily SED-time. In this way, it was possible to assume that time patients were awake without wearing the accelerometer was spent in routine morning and evening sedentary activities, unless spent in PAs that cannot be performed while wearing the accelerometer (e.g., swimming). Total SED-time was then calculated by adding this time to that recorded by the accelerometer. Time spent in non-accelerometer recordable PAs, as reported on the diary, was added to that recorded by the accelerometer, according to the intensity of each activity. Measurements for seven consecutive days were obtained at baseline. Participants were evaluated for physical fitness by assessing cardio-respiratory fitness (as maximal oxygen uptake, VO2max), strength, and flexibility by maximal treadmill exercise test, isometric test, and bending test, respectively [63,64].

### 4.2. Urine Metabolites’Fingerprinting

#### 4.2.1. GC × 2GC-MS/FID Instrument Setup

GC × GC analyses were run with a MPS-2 multipurpose auto-sampler (Gerstel GmbH, Mülheim an der Ruhr, Germany) integrated with an Agilent 6890 GC unit coupled to an Agilent 5975C MS detector (Agilent, Little Falls, DE, USA) operating in EI mode at 70 eV. The GC transfer line was set at 300 °C. An auto-tune option was used and the scan range was set to m/z 50–450 with a scanning rate of 12,500 amu/s to obtain a spectra generation frequency of 20 Hz. The flame ionization detector (FID) was operated as follows: Base temperature 300 °C, H_2_ flow 40 mL/min, air flow 350 mL/min, make-up (N_2_) 20 mL/min, and sampling frequency 200 Hz.

The column set consisted of a first-dimension (1D) column of 30 m × 0.25 mm d_c_ × 0.25 μmd_f_ SE52 (95% polydimethylsiloxane, 5% phenyl) coupled to two second-dimension (2D) columns of equivalent length of 1.4 m × 0.1 mm d_c_ × 0.10 μmd_f_ OV1701 (86% polydimethylsiloxane, 7% phenyl, 7% cyanopropyl). Connections between the primary and the two secondary columns were by a SilFlow™ GC 3 Port Splitter (Trajan Scientific and Medical, Ringwood Victoria, Australia). The secondary column toward the MS detector was connected to a Quick Swap unit (G3185, Agilent, Little Falls, DE, USA) and to an auxiliary electronic pressure controller (EPC) consisting of a one-channel Pneumatics Control Module (G2317A, Agilent, Little Falls, DE, USA). The restrictor capillary in the GC-MS transfer line was of 0.17 m x 0.1 mm d_c_. All columns and capillaries were from Mega (Legnano, Milan, Italy). Helium carrier gas was delivered at constant flow with initial head pressure pi 255.0 KPa and the auxiliary gas (He) for MS outlet pressure correction was delivered at a relative pressure of 39.9 KPa. The estimated split ratio between MS/FID was 50:50. Details on the system configuration have been previously published [36].

Injections for the analysis of both urine samples and n-alkanes for linearretention indices determination *I*^T^s, were by a MPS-2 sampler (Gerstel GmbH, Mülheim an der Ruhr, Germany) under the following conditions: Split/splitless injector, pulsed split mode, split ratio 1/20, injector temperature 290 °C, and injection volume 2 µL. The oven temperature program was 60 °C (1 min) to 300 °C (10 min) at 4.0 °C/min.

The system was equipped with a two-stage KT 2004 loop-type thermal modulator (Zoex Corporation, Houston, TX, USA) cooled with liquid nitrogen and controlled by Optimode™ V.2 (SRA Instruments, Cernusco sul Naviglio (MI), Italy). The hot jet pulse time was set at 350 ms, modulation time was 5 s, and the cold-jet total flow was progressively reduced with a linear function from 30% of mass flow controller (MFC) at initial conditions to 5% at the end of the run. Loop dimensions were chosen on the basis of the expected carrier linear velocities to ensure that two-stage band focusing and release were performed for each modulation stage. The first 0.6 m of the 2Ds were wrapped in the metal slit of the loop-type modulator.

#### 4.2.2. GC-MS Instrument Setup

GC-MS analyses were run for quantitative purposes with the following system configuration: An MPS-2 multipurpose auto-sampler (Gerstel GmbH, Mülheim an der Ruhr, Germany) was integrated with an Agilent 6890 GC unit coupled to an Agilent 5975C MS detector (Agilent, Little Falls, DE, USA) operating in EI mode at 70 eV. The GC transfer line was set at 300 °C. An auto-tune option was used and the scan range was set to m/z 35–550 with a scanning rate of 2500 amu/s.

The column consisted of 30 m × 0.25 mm d_c_ × 0.25 μmd_f_ SE52 (95% polydimethylsiloxane, 5% phenyl) from Mega (Legnano, Milan, Italy). The carrier gas was helium delivered at constant flow (1.0 mL/min) with initial head pressure pi 255.0 KPa.

Injections for the analysis ofurine samples, standard calibration solutions, and n-alkanes for linear retention indices determination IT were by a MPS-2 sampler (Gerstel GmbH, Mülheim an der Ruhr, Germany) under the following conditions: Split/splitless injector, split mode, split ratio 1/20, injector temperature 290 °C, and injection volume 2 µL. The oven temperature program was 80 °C (2 min) to 140 °C at 10.0°C/min then to 240 °C at 4 °C/min up to 290 °C (5 min) at 10 °C/min.

#### 4.2.3. Raw Data Acquisition and GC × GC Data Handling

Data were acquired by Agilent MSD ChemStationver E.02.01.00 and processed using GC Image GC × GC Software version 2.7 (GC Image, LLC Lincoln, Nebraska, USA).

#### 4.2.4. UT Fingerprinting Work-Flow

The 2D data elaboration work-flow is illustrated in Supplementary Figure S1 (SF1). It was optimized and validated in previous studies dealing with complex samples of different natures. It combines untargeted and targeted pattern recognition approaches for the most inclusive fingerprinting.

Steps correspond to the different phases of data elaboration where supervised methods were combined with unsupervised and automated procedures.

The 2D-data processing was by template-matching strategy developed by Reichenbach et al. [65]. In this approach, 2D peak patterns’ metadata (retention times, MS fragmentation, and detector responses) were collected and adopted to establish correspondences between the same analyte across multiple chromatograms. Aligned 2D peaks and 2D peak-regions together with related metadata were made available for comparative purposes and further processing [66,67].

Targeted analysis (Step 1, SF 1) was based on urinary metabolites reliably identified by matching EI-MS fragmentation pattern (NIST MS Search algorithm, ver 2.0, National Institute of Standards and Technology, Gaithersburg, MD, USA, with direct matching threshold 900 and reverse matching threshold 950) with those available in commercial (NIST2014 and Wiley 7n) and in-house databases. Linear retention indices (*I^T^*) along the ^1^D were adopted as additional constraint for positive identification (±10 units).

Untargeted analysis (Step 2, SF 1) was by GC Image Investigator™ R2.7 (GC-Image LLC, Lincoln, Nebraska, USA) and considered 2D peak-regions features [68] above a response threshold of 5000 counts (TIC current). Information about targeted/identified analytes from Step 1was combined to achieve the most inclusive fingerprinting (untargeted/targeted (UT) fingerprinting). This approach aligned all chromatograms using a set of registration peaks to produce a composite chromatogram from which feature peak-regions were extracted. Then, the template with untargeted peak-regions and targeted 2D peaks was aligned with each of the chromatograms of the set for quantitative evaluation. Response data were used for multivariate analysis (MVA).

#### 4.2.5. Quantitative Profiling by GC-MS: Method Performance Verification

Validation of the GC-MS quantitative method followed a previous protocol [31] on a three-weeks-over-two-months period. Method figures of merit considered were: Precision, linearity, accuracy, and limit of quantitation (LOQ) (Commission, 2002; Eurachem, 2014). Precision data (intra- and interweek precision on retention times and 2D peak volumes on analytes’ target ions (Ti)) were evaluated by replicating analyses during the entire period. Linearity was by linear regression analyses within the working range, over at least six different concentration levels.

Calibration solutions for quantitative determination were prepared by mixing single-component standard mother solutions at 10 g/L in suitable solvents (acetone or toluene) and adjusting the final volume up to the required concentration. Each solution was then submitted to derivatization (Section 4.2.6) and directly analyzed. Calibration levels investigated were: 100 mg/L, 40 mg/L, 30 mg/L, 15 mg/L, 10 mg/L, 5 mg/L, 1 mg/L, 0.5 mg/L, and 0.1 mg/L. The 4-fluorophenylalanine, i.e., the internal standard for derivatization quality control was at 10 mg/L.

#### 4.2.6. Reference Materials and Derivatization Procedures

All chemicals were obtained from Merck (Milan, Italy):

(1) Pure standards of n-alkanes (from n-C9 to n-C25) for system evaluation and linear retention index (*I^T^*) determination;
(2) Pure standards for quantitative determinations and/or identity confirmation of pyruvic acid, lactic acid, malonic acid, succinic acid, malic acid, 2-ketoglutaric acid, L-alanine, L-valine, L-leucine, L-proline, glycine, L-threonine, L-tyrosine, L-phenylalanine, xylitol, ribitol, fructose, galactose, glucose, mannitol, myo-inositol, glycerol, creatinine, and the internal standards (ISTDs) 4-fluorophenylalanine (QC for derivatization), and 1,4-dibromobenzene (QC for GC normalization);
(3) Derivatization reagents O-methylhydroxylamine hydrochloride (MOX) and N-methyl-N-(trimethylsilyl)trifluoroacetamide (MSTFA); and
(4) HPLC-grade solvents: Methanol, pyridine, n-hexane, dichloromethane, and toluene.

Urine samples were submitted to a standard derivatization protocol [69] consisting of the following steps: 80 µL of urine and a suitable volume of ISTD (4-fluorophenylalanine solution at 10 g/L) were carefully mixed (Whirlimixer vortex, Fisher Scientific, Loughborough, Leicestershire, UK). Then, the solution was dried under a gentle stream of nitrogen before the addition of 60 µL of MOX. The resulting solution was incubated for 2 h at 60 °C. Next, 60 µL of MSTFA were added and the mixture was incubated at 60 °C for one hour. The resulting sample solution was spiked with 1,4-dibromobenzene at 50 mg/L final concentration and diluted in toluene. Analyses were immediately run by duplicate injections for each sample; samples were randomized within a 24-h time frame after derivatization.

### 4.3. Statistical Analysis

All values in both text and tables were expressed as mean ± sd. Statistical significance was assessed by the Student’s *t* test for parametric data distribution or by Kruskal–Wallis combined to Dunn’s test (*p*) and Bonferroni correction (*^§^p*) for nonparametric data distribution. Testswere performed using the GraphPad Prism version 5.0 for Windows (GraphPad Software, San Diego, California USA) and XLSTAT (Addinsoft, New York, NY, USA).

Untargeted/targeted GC × GC features’ data were processed by Pirouette^®®^ version 4.5 (Infometrix, Inc., Bothell, Washington, USA). Graphical analyses were performed using the R statistical and programming language (3.2.2) (https://cran.r-project.org/) and some add-on packages including ggplot2 and ggpubr.

## Figures and Tables

**Table 1:** Criteria selected for the study groups generation (Group 1: The less sedentary(SED) group, belonging to the first quartile of SED-time, and Group 2: The more sedentary group, belonging to the fourth quartile of SED-time).

	Group 1	Group 2	*p* Value
Sleep duration, h/day	8.04 ± 0.58	8.78 ± 0.95	*** *p* < 0.0001
Sedentary time h/day	11.10 ± 1.02	12.57 ± 0.95	*** *p* < 0.0001
Light-intensity activity h/day	4.63 ± 1.10	2.52 ± 1.14	*** *p* < 0.0001
Mod.—to Vig. intensity activity by accelerometer and diary h/day	0.23 ± 0.07	0.13 ± 0.08	*** *p* < 0.0001

*** *p* < 0.001 Group 2 vs. Group 1.

**Table 2:** Demographic and clinical parameters of study patients.

Clinical Parameters	Group 1	Group 2	*p* Value
Age, years	57.09 ± 7.99	67.98 ± 10.32	*** *p* < 0.0001
Gender: male female	22 (51.2%) 21 (48.8%)	20 (46.5%) 23 (53.5%)	*p* > 0.050
Weight, kg	79.64 ± 15.00	84.87 ± 15.88	*p* > 0.050
BMI, kg/m2	28.56 ± 4.72	30.95 ± 5.21	* *p* = 0.030
Systolic BP, mmHg	136.70 ± 21.06	148.35 ± 22.69	* *p* = 0.039
Diastolic BP, mmHg	82.44 ± 10.03	83.72 ± 16.63	*p* > 0.050
HbA1c, %	7.34 ± 1.53	7.83 ± 1.83	*p* > 0.050
Fasting Plasma Glucose, mg/dl	135.28 ± 49.21	142.65 ± 60.31	*p* > 0.050
Insulin, µU/ml	10.94 ± 10.76	13.33 ± 9.97	*p* > 0.050
HOMA-IR	3.48 ± 2.99	4.41 ± 3.20	*p* > 0.050
Triglycerides, mg/dl	182.40 ± 256.01	177.05 ± 79.64	* *p* = 0.011
Total cholesterol, mg/dl	184.21 ± 36.16	171.63 ± 44.61	*p* > 0.050
HDL cholesterol, mg/dl	48.44 ± 14.93	45.30 ± 13.34	*p* > 0.050
LDL cholesterol, mg/dl	115.88 ± 36.62	100.12 ± 36.63	*p* > 0.050
UKPDS CHD 10-year risk score	14.79 ± 9.48	27.89 ± 17.52	*** *p* < 0.0001
UKPDS FATAL CHD 10-year risk score	9.62 ± 7.39	22.51 ± 16.49	*** *p* < 0.0001
UKPDS STROKE 10-year risk score	7.27 ± 6.29	21.84 ± 16.27	*** *p* < 0.0001
UKPDS FATAL STROKE 10-year risk score	1.14 ± 1.09	3.84 ± 3.34	*** *p* < 0.0001

* *p* < 0.05; *** *p* < 0.001 Group 2 vs. Group 1.

**Table 3:** Metabolites significantly increased in the Group 1 vs. Group 2.

Metabolites	% of Increase Vs. Group 2
FEMALES	
Glycine	312
l-Alanine	85
l-Valine	65
l-Leucine	55
2-Ketoglutaric acid	54
l-Threonine	47
l-Phenylalanine	40
Succinic acid	33
Ribitol	31
Xylitol	25

## Supplementary Materials

The following are available online at https://www.mdpi.com/2218-1989/10/5/205/s1. Table S1: Criteria selected for the study groups’ generation, gender differences. Table S2: Demographic and clinical parameters of study patients, gender differences. Table S3: List of identified urine metabolites for targeted analysis, normalized values over creatinine concentration (mg/L). Figure S1: Schematic diagram illustrating the fingerprinting work-flow.
