# Supplementary material for: Sedentariness and Urinary Metabolite Profile in Type 2 Diabetic Patients, a Cross-Sectional Study
Source: Metabolites. 2020 May 18;10(5):205. doi: 10.3390/metabo10050205 (PMC7281751; doi:10.3390/metabo10050205)
Supplement: Supplementary file 1 [file metabolites-10-00205-s001.zip › metabolites-774041-supplementary file2/metabolites-774041-supplementary file-English Correction.doc]

Supplementary Material

Sedentariness and Urinary Metabolite Profile in Type 2 Diabetic Patients, a Cross-Sectional Study

Elisa Benetti ^1, §,^*, Erica Liberto ^1, §^, Davide Bressanello ^1^, Valentina Bordano ^1^, Arianna C. Rosa ^1^, Gianluca Miglio ^1^, Jonida Haxhi ^2,3^, Giuseppe Pugliese ^2,3^, Stefano Balducci ^2,3,4§^ and Chiara Cordero ^1,§^

^1^	Dipartimento di Scienza e Tecnologia del Farmaco, University of Turin, 10125 Turin, Italy;

^2^	Department of Clinical and Molecular Medicine, ''La Sapienza" University, 00183 Rome, Italy;

^3^	Diabetes Unit, Sant'Andrea Hospital, 00183 Rome, Italy;

^4^	Metabolic Fitness Association, Monterotondo, 00015 Rome, Italy.

*****	Correspondence: elisa.benetti@unito.it;

**^§^**	Both authors contributed equally to this work

**Table S1.** Criteria selected for the study groups’ generation, gender differences.

|  | **GROUP 1** | **GROUP 2** | ***p* Value** |
| --- | --- | --- | --- |
| **Sleep duration, h/day** |  |  |  |
| **male** | 7.95 ± 0.63 | 8.47 ± 0.76 | * *p* = 0.0210 |
| **female** | 8.14 ± 0.51 | 9.06 ± 1.03 | *** *p* = 0.0006 |
| **Time accelerometer worn, h/day** |  |  |  |
| **male** | 15.09 ± 0.72 | 14.55 ± 0.55 | * *p* = 0.0107 |
| **female** | 15.04 ± 0.59 | 14.00 ± 1.19 | *** *p* = 0.0008 |
| **Time accelerometer not worn, h/d** |  |  |  |
| **male** | 0.88 ± 0.43 | 0.99 ± 0.57 | *p* > 0.050 |
| **female** | 0.82 ± 0.32 | 0.84 ± 0.37 | *p* > 0.050 |
| **Sedentary time, h/day** |  |  |  |
| **male** | 10.93 ± 1.10 | 12.46 ± 1.07 | *** *p* < 0.0001 |
| **female** | 11.27 ± 0.92 | 12.66 ± 0.84 | *** *p* < 0.0001 |
| **Light-intensity activity, h/day** |  |  |  |
| **male** | 4.85 ± 1.28 | 2.90 ± 1.11 | *** *p* < 0.0001 |
| **female** | 4.39 ± 0.84 | 2.18 ± 1.08 | *** *p* < 0.0001 |
| **Mod. to Vig. intensity activity by accelerometer and diary, h/day** | | | |
| **male** | 0.27 ± 0.05 | 0.17 ± 0.07 | *** *p* < 0.0001 |
| **female** | 0.20 ± 0.07 | 0.09 ± 0.07 | *** *p* < 0.0001 |

**Table S2.** Demographic and clinical parameters of study patients, gender differences.

|  | **GROUP 1** | **GROUP 2** | ***p* Value** |
| --- | --- | --- | --- |
| **Age, years** |  |  |  |
| **male** | 56.27 ± 5.85 | 67.00 ± 11.39 | *** p = 0.0004 |
| **female** | 57.95 ± 9.83 | 68.83 ± 9.48 | *** p = 0.0007 |
| **Gender :** |  |  |  |
| **male** | 22 (51.2%) | 20 (46.5%) | *p* > 0.050 |
| **female** | 21 (48.8%) | 23 (53.5%) | *p* > 0.050 |
| **Weight, kg** |  |  |  |
| **male** | 84.00 ± 13.02 | 85.97 ± 10.85 | *p* > 0.050 |
| **female** | 75.08 ± 15.88 | 81.55 ± 15.25 | *p* > 0.050 |
| **BMI, kg/m^2^** |  |  |  |
| **male** | 28.16 ± 3.92 | 29.43 ± 4.75 | *p* > 0.050 |
| **female** | 29.00 ± 5.50 | 32.28 ± 5.34 | p > 0.050 |
| **Systolic BP, mmHg** |  |  |  |
| **male** | 139.50 ± 24.06 | 145.45 ± 18.32 | *p* > 0.050 |
| **female** | 133.76 ± 17.50 | 150.87 ± 26.05 | * *p =* 0.0152 |
| **Diastolic BP, mmHg** |  |  |  |
| **male** | 85.05 ± 10.89 | 86.70 ± 22.03 | *p* > 0.050 |
| **female** | 79.71 ± 8.47 | 81.13 ± 9.67 | *p* > 0.050 |
| **Family history of diabetes** | 72% | 91% | *p* > 0.050 |
| **HbA_1c_, %** |  |  |  |
| **male** | 7.69 ± 1.65 | 7.69 ± 1.73 | *p* > 0.050 |
| **female** | 6.97 ± 1.33 | 7.95 ± 1.94 | *p* > 0.050 |
| **Fasting plasma glucose, mg/dl** | |  |  |
| **male** | 137.23 ± 46.87 | 135.70 ± 47.51 | *p* > 0.050 |
| **female** | 133.24 ± 52.64 | 148.70 ± 70.09 | *p* > 0.050 |
| **Insulin, µU/ml** |  |  |  |
| **male** | 9.94 ± 8.73 | 12.50 ± 9.82 | *p* > 0.050 |
| **female** | 12.04 ± 12.79 | 14.07 ± 10.25 | *p* > 0.050 |
| **HOMA-IR** |  |  |  |
| **male** | 3.35 ± 2.74 | 4.12 ± 3.27 | *p* > 0.050 |
| **female** | 3.65 ± 3.31 | 4.68 ± 3.19 | *p* > 0.050 |
| **Triglycerides, mg/dl** |  |  |  |
| **male** | 210.64 ± 346.82 | 171.45 ± 99.96 | *p* > 0.050 |
| **female** | 152.81 ± 97.71 | 181.91 ± 58.52 | * *p* = 0.039 |
| **Total cholesterol, mg/dl** |  |  |  |
| **male** | 176.45 ± 40.97 | 166.05 ± 44.69 | *p* > 0.050 |
| **female** | 192.33 ± 29.13 | 176.48 ± 44.96 | *p* > 0.050 |

**Table S2:** Demographic and clinical parameters of study patients, gender differences. (Continue)

|  | **GROUP 1** | **GROUP 2** | **p value** |
| --- | --- | --- | --- |
| **HDL cholesterol, mg/dl** |  |  |  |
| **male** | 44.64 ± 15.48 | 41.95 ± 12.98 | *p* > 0.050 |
| **female** | 52.43 ± 13.56 | 48.22 ± 13.24 | *p* > 0.050 |
| **LDL cholesterol, mg/dl** |  |  |  |
| **male** | 111.45 ± 43.48 | 98.30 ± 39.87 | *p* > 0.050 |
| **female** | 120.52 ± 28.04 | 101.70 ± 34.39 | p > 0.050 |
| **UKPDS CHD** |  |  |  |
| **10-year risk score** |  |  |  |
| **male** | 18.88 ± 9.54 | 35.95 ± 19.50 | *** *p* = 0.0007 |
| **female** | 10.51 ± 7.44 | 20.88 ± 12.14 | *** *p* = 0.0006 |
| **UKPDS FATAL CHD** |  |  |  |
| **10-year risk score** |  |  |  |
| **male** | 12.01 ± 7.44 | 28.30 ± 18.92 | *** *p* = 0.0010 |
| **female** | 7.11 ± 6.60 | 17.49 ± 12.37 | *** *p* = 0.0003 |
| **UKPDS STROKE** |  |  |  |
| **10-year risk score** |  |  |  |
| **male** | 6.86 ± 4.68 | 24.88 ± 18.53 | *** *p* = 0.0001 |
| **female** | 7.69 ± 7.73 | 19.21 ± 13.90 | *** *p* = 0.0008 |
| **UKPDS FATAL STROKE** | |  |  |
| **10-year risk score** |  |  |  |
| **male** | 1.10 ± 0.88 | 4.05 ± 3.11 | *** *p* < 0.0001 |
| **female** | 1.17 ± 1.31 | 3.66 ± 3.59 | *** *p* = 0.0006 |

**Table S3. List of identified urine metabolites for targeted analysis, normalized values over creatinine concentration (mg/L).**

Available as Excel file uploaded separately.

**Figure SF1: Schematic diagram illustrating the fingerprinting work-flow (modified from [30]).**

|  | © 2020 by the authors. Submitted for possible open access publication under the terms and conditions of the Creative Commons Attribution (CC BY) license (http://creativecommons.org/licenses/by/4.0/). |
| --- | --- |
